# LINC01503/miR-342-3p facilitates malignancy in non-small-cell lung cancer cells via regulating LASP1

**DOI:** 10.1186/s12931-020-01464-3

**Published:** 2020-09-16

**Authors:** Qiming Shen, Yanbin Sun, Shun Xu

**Affiliations:** Thoracic Surgery, The First Hospital of China Medical University, No.155 North Nanjing Road, Heping Area, Shenyang, 110001 China

**Keywords:** LINC01503, miR-342-3p, Non-small-cell lung cancer, LASP1

## Abstract

**Background:**

Non-small cell lung cancer (NSCLC) is one of the major types of lung cancer, which is a prevalent human disease all over the world. LncRNA LINC01503 is a super-enhancer-driven long non-coding RNA that is dysregulated in several types of human cancer. However, its role in NSCLC remains unknown.

**Methods:**

Thirty NSCLC patients were recruited between April 2012 and April 2016. Luciferase reporter assay, qRT-PCR, Cell Counting Kit-8 (CCK-8), Transwell migration assay, RNA pull-down assay, western blotting, 5-ethynyl-29-deoxyuridine (EdU) assays, and flow cytometry were utilized to characterize the roles and relationships among LINC01503, miR-342-3p, and LASP1 in NSCLC. The transplanted mouse model was built to examine their biological functions in vivo.

**Results:**

We demonstrated that the expression of lncRNA LINC01503 and LIM and SH3 domain protein 1 (LASP1) were upregulated and miR-342-3p was downregulated in NSCLC samples and cell lines. Functional experiments revealed that inhibiting the expression of LINC01503 or over-expression of miR-342-3p inhibited NSCLC growth and metastasis both in vitro and in vivo. In addition, LINC01503 could bind to miR-342-3p and affect the expression of LASP1.

**Conclusion:**

These results provide a comprehensive analysis of the roles of LINC01503 as a competing endogenous RNA (ceRNA) in NSCLC progression.

## Background

Non-small cell lung cancer (NSCLC) is one of the major types of lung cancer, which is a quite prevalent human disease all over the world [1]. Extensive efforts have been made to discover new therapeutic targets for the treatment of NSCLC [2, 3]. However, as the early symptoms of NSCLC are not obvious, a large portion of NSCLC patients was diagnosed at late stages, with high malignancy and low treatment opportunities [1, 4]. The current overall 5-year survival rate of NSCLC patients still remains unsatisfactory [5]. In this study, we focused to understand the pathogenesis of NSCLC, as well as to investigate new therapeutic targets based on the tumorigenesis of NSCLC.

Long noncoding RNAs (lncRNAs, > 200 nt) are a major subtype of non-coding RNAs that cannot translate to proteins [6, 7]. A large number of lncRNAs have been discovered to play vital roles in tumorigenesis, with an unignorable impact in many biological procedures such as cell proliferation, cell cycle, cell death, as well as tumor metastasis [8, 9]. Previous studies have revealed the expression profiles of several lncRNAs that participate closely in the tumorigenesis of NSCLC, including lncRNA PVT1 [10], lncRNA AFAP1-AS1 [11], MEG3 [12], and so on. LncRNA LINC01503 is a super-enhancer-driven lncRNA that is dysregulated in several types of human cancer. For example, it was reported that lncRNA LINC01503, regulated by TP63, is over-expressed and oncogenic in squamous cell carcinoma [13]. However, its role in NSCLC remains unknown.

As another major subtype of non-coding RNA, microRNAs (miRNAs, ~ 20 nt) exert tumor-suppressive functions in many types of human cancer [14, 15]. For NSCLC, miRNAs are usually working together with specific lncRNAs and down-stream factors to regulate their progression and metastasis [16]. MiR-342-3p was reported to target RAP2B to suppress proliferation and invasion of non-small cell lung cancer cells [17]. One report revealed that miR-342-3p suppresses cell proliferation, migration, and invasion by targeting forkhead box protein M1 (FOXM1) in human cervical cancer. In our preliminary experiments, Starbase predicted that lncRNA LINC01503 could target miR-342-3p. LIM and SH3 domain protein 1 (LASP1) is a potential negative predictor of NSCLC [18]. It has been proved to be the down-stream factor of miR-203 [19] and miR-29a [20] in the suppression or proliferation of human NSCLC. Therefore, this study was carried out to investigate the associations among lncRNA LINC01503, miR-342-3p, and LASP1 in the development and progression of NSCLC.

## Methods

### Patients and samples

A total of 30 NSCLC patients were recruited in the First Hospital of China Medical University, P.R. China between April 2012 and April 2016. Their ages ranged from 41 to 69 years old, with an average age of 53 years old. These patients did not receive any treatment before the surgeries. After sections, NSCLC tissues and matched normal tissue samples were frozen in liquid nitrogen and stored at − 80 °C. This study was approved by the Ethics Committee of the First Hospital of China Medical University, P.R. China. All the patients signed written informed consent.

### Cells culture

NSCLC cell lines A549, SPC-A1, H1299, H1650, H1975, and PC-9 and normal cell line of 16HBE were provided by Institute of Biochemistry and Cell Biology, Chinese Academy of Sciences. Cells were cultured in Dulbeccos modified Eagles medium (DMEM) supplemented with 10% fetal bovine serum (FBS; Invitrogen), 100 U/mL penicillin and 100 mg/mL streptomycin at 37 °C in a humidified incubator with 5% CO_2_.

### Plasmid construction and transfection

MiR-342-3p-mimics, miR-342-3p-inhibitor, miR-NC and anti-miR-NC were provided by RiboBio, China. Sh-LINC01503 and sh-NC were provided by Genepharma, Shanghai. The sequences were: LINC01503, sh-1 target 5′-TCTGACAAGTGTGTACCTA-3′; sh-LINC01503, 5′-AATTCTCCGAACGTGTCACGT-3′. LASP1 cDNA was cloned into pcDNA3.1 (Invitrogen, USA). LASP1-WT (wild-type) or LSAP1-MUT (mutant) had amplification using PCR, and transfection to A549 cells with Lipofectamine 2000 (Invitrogen, USA).

### Luciferase reporter assays

LINC01503 and LASP1 wild-type or mutant binding miR-342-3p were cloned to pMIR Basic vector (OBiO Biology, Beijing) to generate pMIR-REPOR-LINC01503-wt or pMIR-REPOR-LINC01503-mt, respectively. After being cultivated in 24-well plates for 24 h, A549 cells were transfected with miR-342-3p mimics or control (GenePharma, China) and co-transfected with pMIR-REPOR-NC or constructed recombinant luciferase vectors by Lipofectamine 3000 (Invitrogen, USA). Luciferase activities were detected and compared with Renilla luciferase activity, which is an internal control for normalization using dual-luciferase reporter assays 48 h after transfection.

### qRT-PCR

RNAs were extracted by TRIzol (Invitrogen, USA). Reverse transcription of RNAs to cDNA was performed using Reverse Transcription Kit (Tiangen, China). qRT-PCR was performed using SYBR (Takara, Japan) on an ABI7300 Real-Time PCR System (Applied Biosystems, USA). U6 and GAPDH were used as endogenous control. The expression levels were calculated using the 2^-△△Ct^ method [21]. The primers used were:

LINC01503, 5′-GGGACGGAGACAAATGACGG-3′ (forward) and 5′-GCAGGCTCCCTGACACGTA-3′ (reverse);

U6, 5′-AACGAGACGACGACAGAC-3′ (forward) and 5′-GCAAATTCGTGAAGCGTTCCATA-3′ (reverse);

GAPDH, 5′-ATGTTGCAACCGGGAAGGAA-3′ (forward) and 5′-AGGAAAAGCATCACCCGGAG-3′ (reverse);

miR-342-3p, 5′-GGGTCTCACACAGAAATCGC-3′ (forward) and 5′-CAGTGCGTGTCGTGGAGT-3′ (reverse);

LASP1, 5′-TGCGGCAAGATCGTGTATCC-3′ (forward) and 5′-GCAGTAGGGCTTC TTCTCGTAG-3′ (reverse).

### Mouse model

Male BALB/c nude mice (20–22 g) were provided by Animal Center of the Chinese Academy of Science. A total of 2,000,000 cells in transfection with NC or sh-LINC01503 or miR-342-3p-inh were injected to the mice (*n* = 5). Tumor sizes were detected every week. After 28 d, tumor volumes were calculated according to length x (width^2^/2). The tumor weights were measured. Animal experiments were conducted according to Guide for the Care and Use of Laboratory Animals of the National Institutes of Health and Ethics Committee of the First Hospital of China Medical University.

### Cell counting Kit-8 (CCK-8)

In each well of the 96-well plate, 5000 cells were seeded and cultured. LINC01503 or control was transfected to cells. At 1, 2, 3 and 4 d after transfection, 10 μl CCK-8 solution was added to the wells. The absorbance of each plate was examined at 450 nm after 1 h of incubation.

### Transwell migration assay

A total of 100,000 cells were seeded on 24-well Transwell plate. The lower chamber (coated with Matrigel) was placed with medium and 20% FBS. After 1 d of incubation, cells were fixed by 4% paraformaldehyde and stained with crystal violet. The migration assay was conducted without coating the membranes with Matrigel. Invaded cells were recorded and counted by a microscope.

### RNA pull-down assay

Biotin-labeled RNAs were transcribed with AmpliScribe T7-Flash Biotin-RNA Tran-scription Kit (Epicentre) and incubated with RNase-free DNase I and purified with RNeasy Mini Kit (Qiagen). The lambda transcript was generated with the control plasmid provided by the Transcription Kit. Biotinylated RNA supplied with RNA structure buffer (10 mM Tris pH 7, 0.1 M KCl and10 mM MgCl_2_) was heated to 90 °C for 2 min, incubated on ice for 2 min and then shifted to room temperature (RT) for 20 min. Biotinylated RNAs were mixed with purified proteins and incubated for 1 h. Then, the RNAs were incubated by Streptavidin Mag Sepharose (GE Healthcare) for 1 h. After washing, the pull-down assay was measured by PCR.

### Western blotting

Cells were lysed and separated by SDS-PAGE. Proteins were transferred to PVDF membranes (Millipore, USA), which were blocked by 5% skimmed milk. Then, the membranes were incubated by anti-LASP1 (1:1000; Abcam, UK) and anti-GAPDH (1:1000; Cell Signal Technology, USA) at 4 °C overnight. Next, the membranes were treated by HRP-labeled anti-IgG for 1 h. Results were measured by the Bio-Rad system.

### EdU assays

Cells were digested and seeded them into 96 well plates 1 d post-transfection. After 2 d, the EdU proliferation assays were conducted by EdU Apollo (RiboBio, China). The proliferated cells (EdU positive) were observed under the microscope.

### Flow cytometry

Cell apoptosis was measured by Annexin V (Biosea, China) at 2 d post-transfection and Cell Quest (BD, USA). For cell cycle, 1,000,000 cells were collected, fixed, stained by propidium iodide, and measured by flow cytometry.

### Immunohistochemical staining

Samples were formalin fixed and paraffin embedded, and were subjected to immunohistochemical evaluation for Ki-67, according to the method described in previous literature [22].

### Statistical analyses

Statistical analyses were conducted by SPSS 19. Data were shown as mean ± SD and comparisons were performed by student’s t-test (2 groups) or ANOVA test (> 2 groups). The χ2 test was used to analyze the relationship between the expression of LINC01503 and LASP1. Kaplan-Meier and log-rank were utilized to generate and analyze survival curves. *P* < 0.05 was considered as significant differences.

## Results

### The expression of LINC01503 was upregulated in NSCLC patients and NSCLC cells

**Fig. 1:**
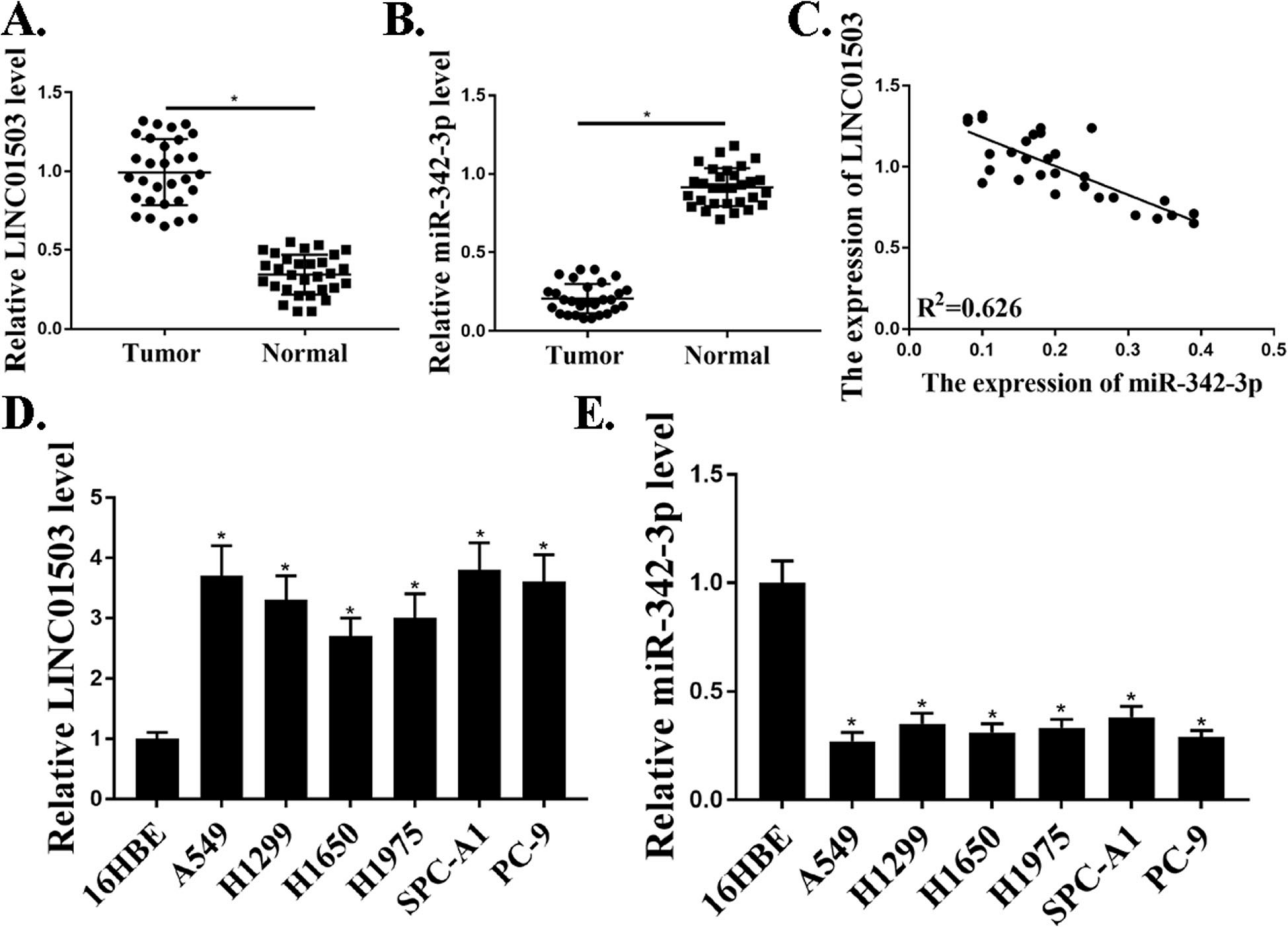
Up-regulation of LINC01503 in NSCLC patients and NSCLC cells. **a.** Relative expression of LncRNA-LINC001503 in NSCLC samples and adjacent normal samples (*n* = 30). **b**. Relative expression of miR-342-3p in NSCLC samples and adjacent normal samples (*n* = 30). **c**. The correlation between LINC01503 and miR-342-3p levels was measured. **d**. The expression levels of LINC01503 in NSCLC cells. **e**. The expression levels of miR342-3p in NSCLC cells. *N* = 3, *p < 0.05

To identify the roles of LINC01503 in NSCLC progression, qRT-PCR analysis was used to investigate the expression of LINC01503 in 30 pairs of NSCLC samples compared to that in adjacent normal samples. Our results revealed that the expression level of LINC01503 in tumor samples were markedly elevated compared with those in the corresponding normal samples (*P*<0.05) (Fig. 1a). However, the expression levels of miR-342-3p were significantly lower in tumor samples compared with that in normal control samples (*P*<0.05) (Fig. 1b). Moreover, the expression of miR-342-3p was negatively related to the expression of LINC01503 in NSCLC patients (Fig. 1c). We also examined the expression of LINC01503, miR-342-3p in NSCLC cells. As shown in Fig. 1d, e, the expression levels of LINC01503 were also markedly increased and the expression levels of miR-342-3p were decreased in NSCLC cells compared with that in control 16HBE cells (*P*<0.05). These results demonstrated that the expression of LINC01503 was upregulated in NSCLC patients and NSCLC cells.

### Knockdown of LINC01503 suppressed proliferation, migration, and invasion of NSCLC cells in vitro

**Fig. 2:**
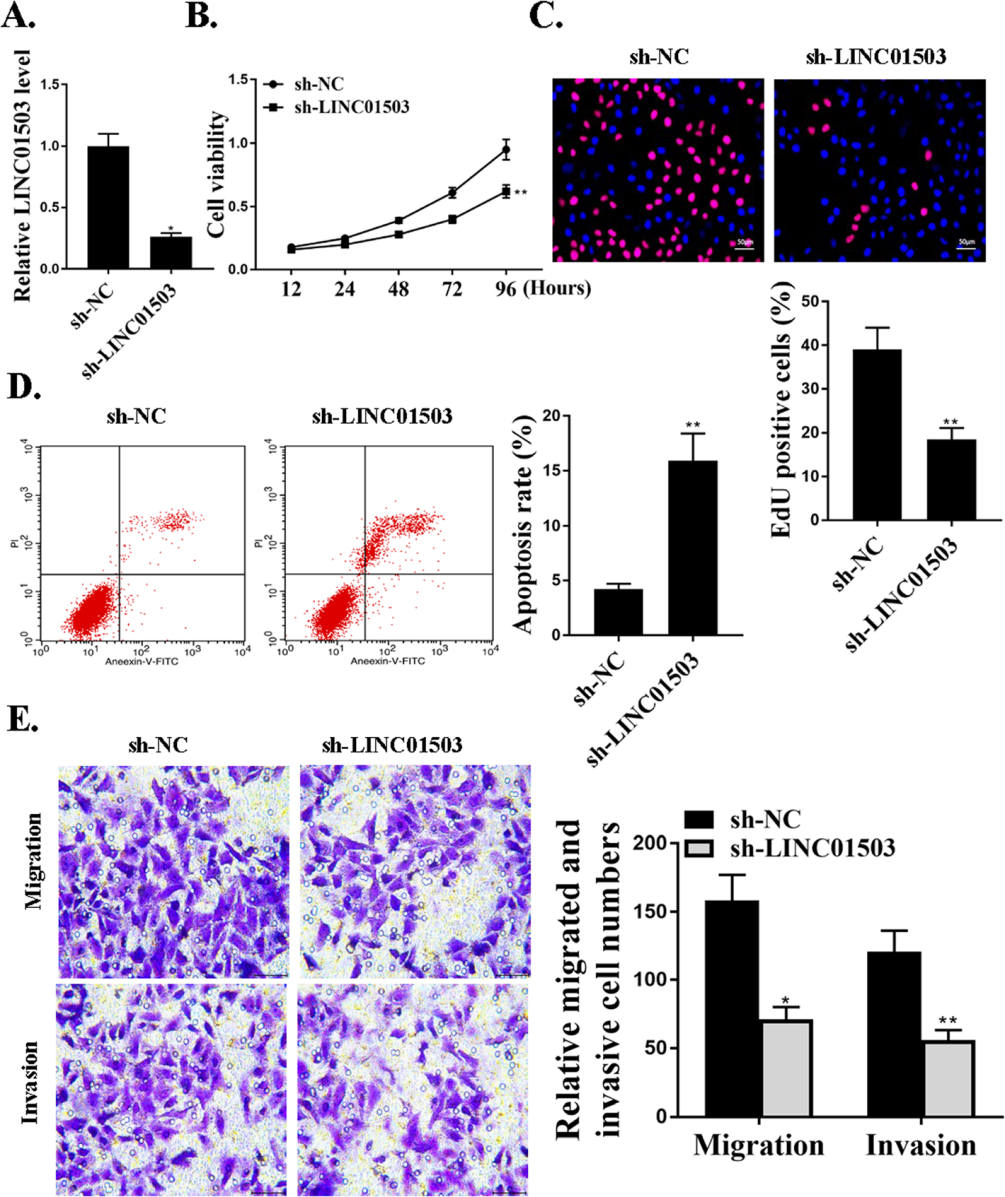
LINC01503 promotes proliferation, migration, and invasion of NSCLC cells in vitro. **a**. The expression levels of LINC01503 in cells transfected with sh-NC or sh-LINC01503. **b**. CCK8 assay for cell apoptosis rate in cells transfected with sh-NC or sh-LINC01503. **c**. Edu staining for cell viability in cells transfected with sh-NC or sh-LINC01503. For the colors, blue indicates Hoechst, and red indicates Edu. **d**. Cytoflow to examine cell apoptosis in cells transfected with sh-NC or sh-LINC01503. **e**. Transwell assays for cell invasive and migrative abilities in cells transfected with sh-NC or sh-LINC01503. *N* = 3, *p < 0.001, **p < 0.05

To investigate the role of LINC01503 in NSCLC progression, knockdown experiments were utilized. Lentiviruses expressing specific shRNA targeting LINC01503 (sh-LINC01503) and negative control shRNA (sh-NC) were used in cells. The reduced expression levels of LINC01503 in these cells transfected with sh-LINC01503 were confirmed by qRT-PCR (Fig. 2a). The effect of LINC01503 on cell proliferation was evaluated by CCK-8 assay and Edu assay, and the data revealed that sh-LINC01503 inhibition significantly reduced cell proliferation compared with that in the control groups (*P*<0.05) (Fig. 2b and c). Transwell assay further revealed a notable reduction in the number of migrated (*P*<0.05) and invaded cells (*P*<0.01) in LINC01503 shRNA transfection group compared to the negative control shRNA transfection group (Fig. 2e). As shown in Fig. 2d, sh-LINC01503 transfection significantly increased cell apoptosis compared with that in sh-NC transfection group (*P*<0.01). It is obvious that knockdown of LINC01503 could suppress the proliferation, migration, and invasion of NSCLC cells in vitro.

### MiR-342-3p mediated proliferation, migration, and invasion of NSCLC cells in vitro

**Fig. 3:**
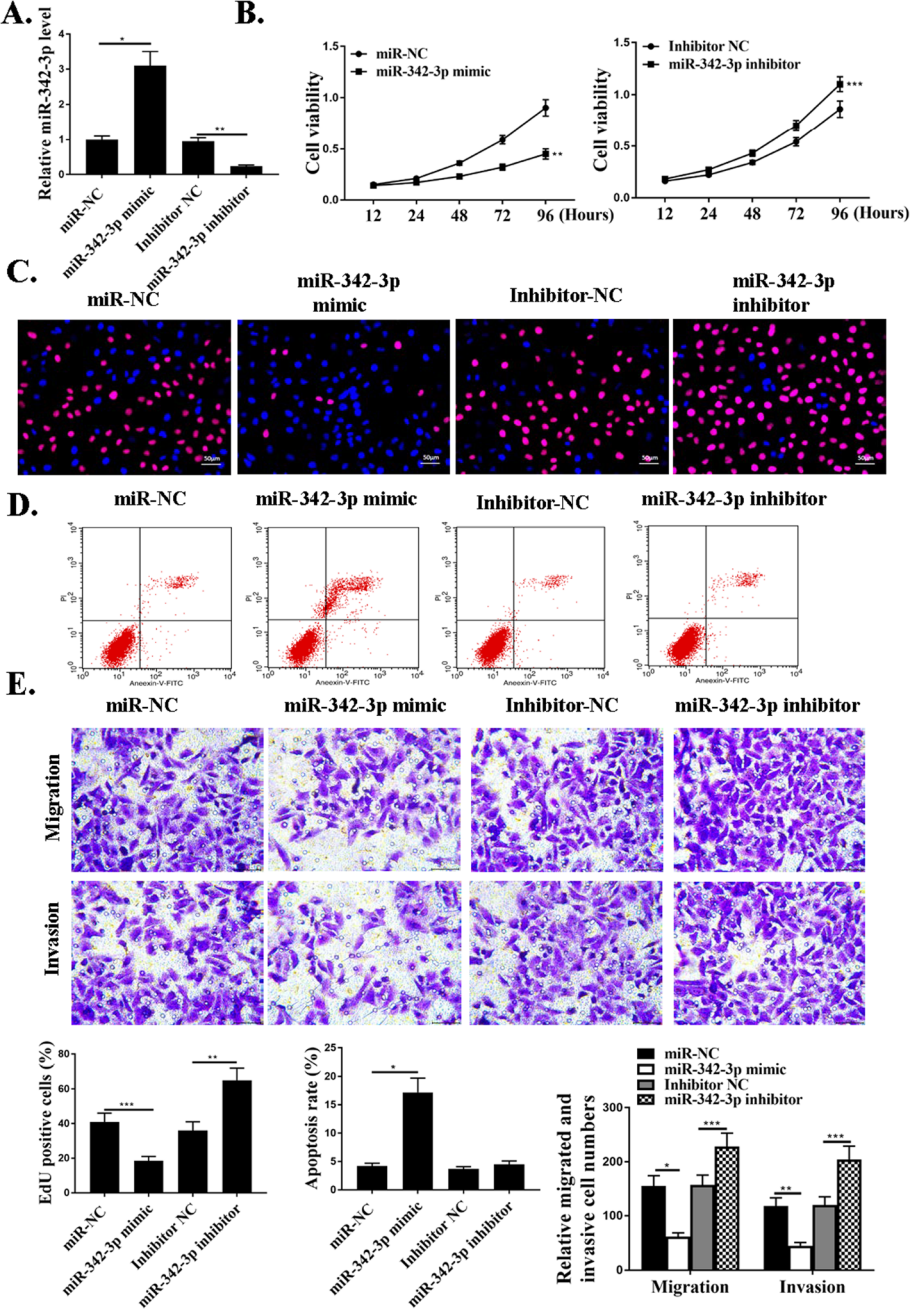
MiR-342-3p mediates proliferation, migration, and invasion of NSCLC cells in vitro. **a**. The expression levels of miR-342-3p in cells transfected with miR-NC, miR-342-3p-mimic, inhibitor NC, or miR-342-3p inhibitor. **b**. CCK8 assay for cell viabilities in cells transfected with miR-NC, miR-342-3p-mimic, inhibitor NC, or miR-342-3p inhibitor. **c**. Edu staining in cells transfected with miR-NC, miR-342-3p-mimic, inhibitor NC, or miR-342-3p inhibitor. For the colors, blue indicates Hoechst, and red indicates Edu. **d**. Cytoflow to examine cell apoptosis in cells transfected with miR-NC, miR-342-3p-mimic, inhibitor NC, or miR-342-3p inhibitor. **e**. Transwell assays for cell invasive and migrative abilities in cells transfected with miR-NC, miR-342-3p-mimic, inhibitor NC, or miR-342-3p inhibitor. *N* = 3, *p < 0.001,**p < 0.01, ***p < 0.05

To understand the functional roles of miR-342-3p in NSCLC progression, knockdown and over-expression experiments were performed. MiR-342-3p mimic or miR-342-3p inhibitor and negative control NC were used in cells. The expression of miR-342-3p in these cells was detected by qRT-PCR (Fig. 3a). The effect of miR-342-3p on cell proliferation was evaluated by CCK-8 assay and EdU assay, and it revealed that transfection of miR-342-3p mimic significantly reduced cell proliferation compared with that in the control groups (*P*<0.01) (Fig. 3b and c). As shown in Fig. 3d, miR-342-3p mimic transfection significantly increased cell apoptosis compared with that in the control groups (*P*<0.01). Transwell assay was further utilized to assess the influence of miR-342-3p on the migration and invasion. The data revealed a notable reduction in the number of migrated and invaded cells in miR-342-3p mimic transfection group compared with that in the negative control groups (*P*<0.001) (Fig. 3e). In summary, miR-342-3p could mediate the proliferation, migration, and invasion of NSCLC cells in vitro.

### MiR-342-3p was a target of LINC01503

**Fig. 4:**
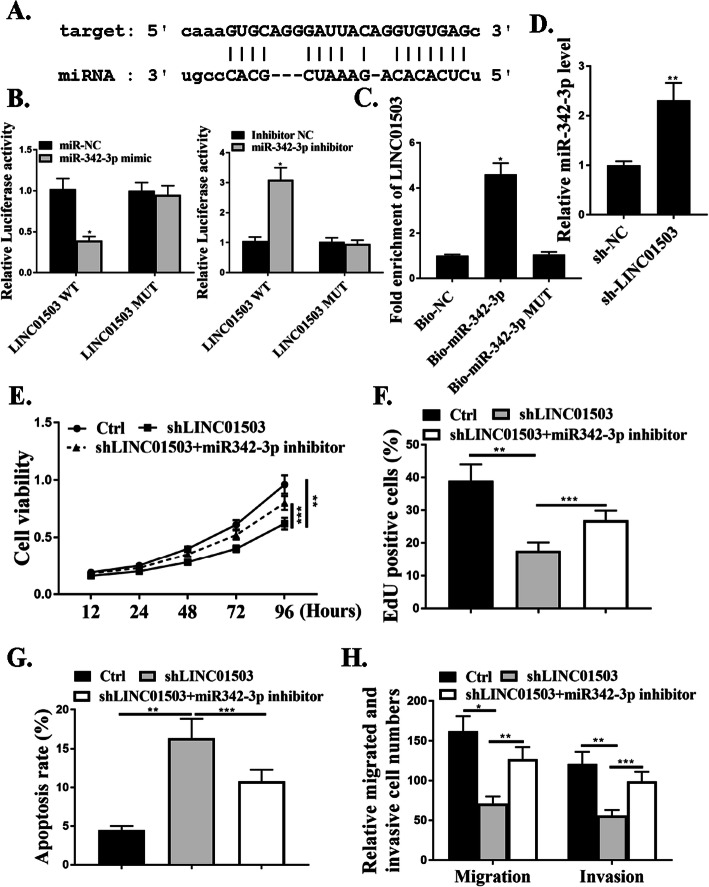
MiR-342-3p is a target of LINC01503 and LASP1 is a target of miR-342-3p. **a.** The putative target sequence for miR-342-3p on the 3′-UTR of LINC01503. **b**. The luciferase activity was detected by luciferase reporter assay. **c**. RNA pull-down assay. **d**. The expression levels of miR-342-3p in cells. **e**. CCK8 assay. **f**. Edu staining. **g**. Cytoflow to examine cell apoptosis. **h**. Transwell assay. *N* = 3, *p < 0.001, **p < 0.01. ***p < 0.05

We next explored the underlying mechanism of how LINC01503 regulates NSCLC progression. The starBase online tool was used to explore the association of LINC01503 and microRNAs. We noticed that miR-342-3p, a tumor suppressor microRNA, was predicted to interact with LINC01503 (Fig. 4a). As shown in Fig. 4b, miR-342-3p markedly inhibited the luciferase activities in cells in transfected with the LINC01503-WT (*P*<0.05), but did not affect the luciferase activities transfected with LINC01503-MUT (*P*>0.05). The results from RNA pull-down assay showed a markedly elevated fold enrichment of LINC01503 using Bio-miR-342-3p, compared with the assays using NC-bio or Bio-miR-342-3p MUT (*P*<0.05) (Fig. 4c). Results also showed that the expression levels of miR-342-3p were significantly increased after transfection of sh-LINC01503 (*P*<0.01) (Fig. 4d).

To understand the functional roles of LINC01503/miR-342-3p in NSCLC progression, knockdown experiments were utilized. Lentiviruses expressing specific shRNA targeting LINC01503 (sh-LINC01503) and negative control shRNA (sh-NC) were used in cells. The effect of LINC01503 on cell proliferation was evaluated by CCK-8 assay and EdU assay, and the data revealed that sh-LINC01503 transfection reduced cell proliferation compared with that in the control group cells (*P*<0.01), and this effect was attenuated by co-transfection with miR-342-3p inhibitor (*P*<0.001) (Fig. 4e and f). Transwell assay was further utilized to assess the influence of LINC01503 on the migration and invasion. The data revealed a notable reduction in the number of migrated and invaded cells in transfection with sh-LINC01503 compared with that in the negative control shRNA group, and this effect was attenuated by co-transfection with miR-342-3p inhibitor (*P*<0.01, *P*<0.001) (Fig. 4h). As shown in Fig. 4g, sh-LINC01503 promoted cell apoptosis and this effect was attenuated by co-transfection with miR-342-3p inhibitor (*P*<0.001). These results indicated that miR-342-3p was a target of LINC01503.

### LASP1 was a target of miR-342-3p

**Fig. 5:**
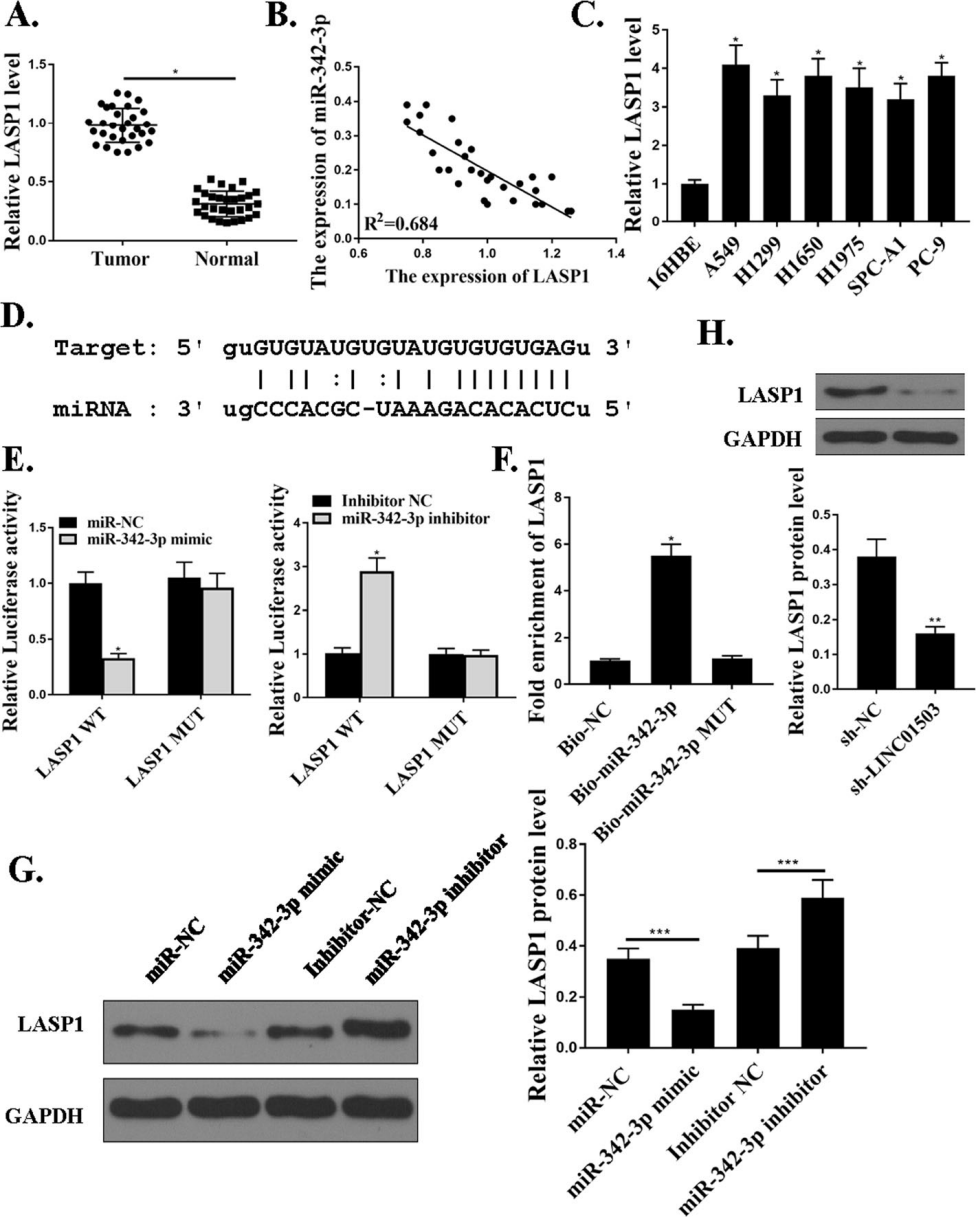
LASP1 is the target ofmiR-342-3p. **a**. Relative expression of LASP1 in NSCLC samples and adjacent normal samples (*n* = 30). **b**. The correlation between miR-342-3p and LASP1 levels was measured. **c**. The expression levels of miR342-3p in NSCLC cells. H. The expression levels of LASP1 in NSCLC cells. **d**. The putative target sequence for miR-342-3p on the 3′-UTR of LASP1. **e** The luciferase activity was detected by luciferase reporter assay. **f**. RNA pull-down assay. **g**. and **h**. The LASP1 protein expression. *N* = 3, *p < 0.001, **p < 0.01. ***p < 0.05

The expression of LASP1(LIM and SH3 domain protein 1) was upregulated in tumor samples (Fig. 5a). Moreover, the expression of miR-342-3p was negatively related with that of LASP1 in NSCLC patients (Fig. 5b). We also examined the expression of LASP1 in NSCLC cells. As shown in Fig. 5c, the expression levels of LASP1 were also significantly increased in NSCLC cells compared with that in 16HBE cells (*P*<0.05). Next, we noticed that miR-342-3p, a tumor suppressor microRNA, was predicted to interact with LASP1 (Fig. 5d). As shown in Fig. 5e, the expression of miR-342-3p markedly inhibited the luciferase activities in cells in transfection with LASP1-WT (*P*<0.05), but did not affect the luciferase activity in cells in transfection with LASP1-MUT (*P*>0.05). RNA pull-down assays pointed out a markedly elevated expression levels of LASP1 using -miR-342-3p compared to that with the control (NC-bio) or hsa-mir-miR-342-3p probes (*P*<0.05) (Fig. 5f). In addition, miR-342-3p and LINC01503 regulated the expression of LASP1 (Fig. 5g and h). As a result, we established that LASP1 was a target of miR-342-3p.

### MiR-342-3p/LASP1 axis mediated proliferation, migration, and invasion of NSCLC cells in vitro

**Fig. 6:**
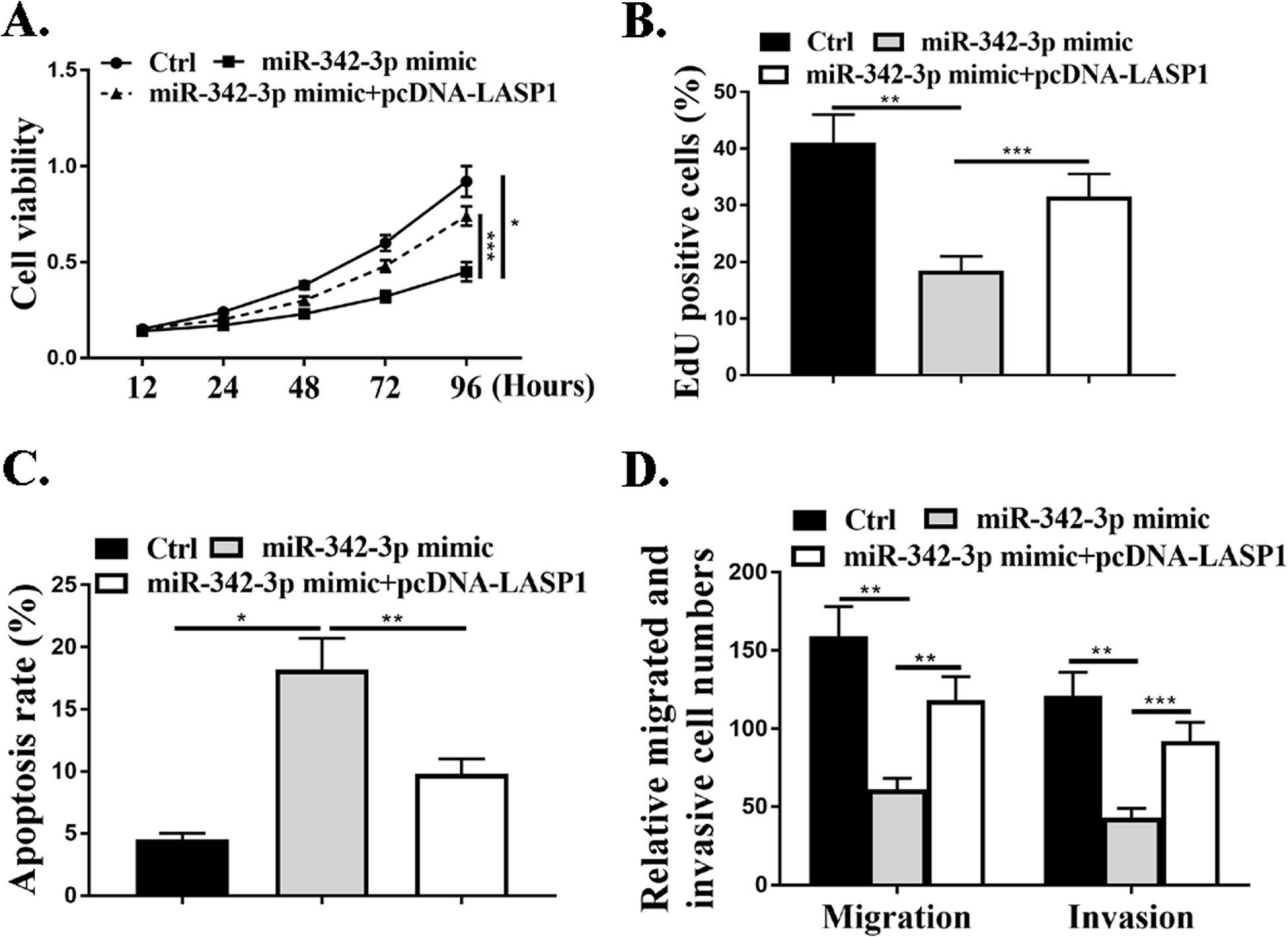
MiR-342-3p/LASP1 axis mediated proliferation, migration, and invasion of NSCLC cells in vitro. **a**. CCK8 assay. **b**. Edu staining. **c**. Cytoflow to examine cell apoptosis. **d**. Transwell assay. *N* = 3, *p < 0.001, **p < 0.01, ***p < 0.05

The effect of miR-342-3p on cell proliferation was evaluated by CCK-8 assay and EdU assay and the data revealed that miR-342-3p mimic reduced cell proliferation compared with that in the control cells (*P*<0.05), and this effect was attenuated by co-transfection with pcDNA-LASP1 (*P*<0.001) (Fig. 6a and b). Transwell assay results revealed a notable reduction in the number of migrated and invaded cells in transfection with miR-342-3p mimic relative to the negative control group (*P*<0.01) and this effect was attenuated by co-transfection with pcDNA-LASP1(*P*<0.01, *P*<0.001) (Fig. 6d). As shown in Fig. 6c, miR-342-3p mimic promoted cell apoptosis and this effect was attenuated by co-transfection with pcDNA-LASP1 (*P*<0.01). The above data revealed that miR-342-3p/LASP1 axis could mediate the proliferation, migration, and invasion of NSCLC cells in vitro.

### LINC01503/miR-342-3p promotes NSCLC cell tumor growth in vivo

**Fig. 7:**
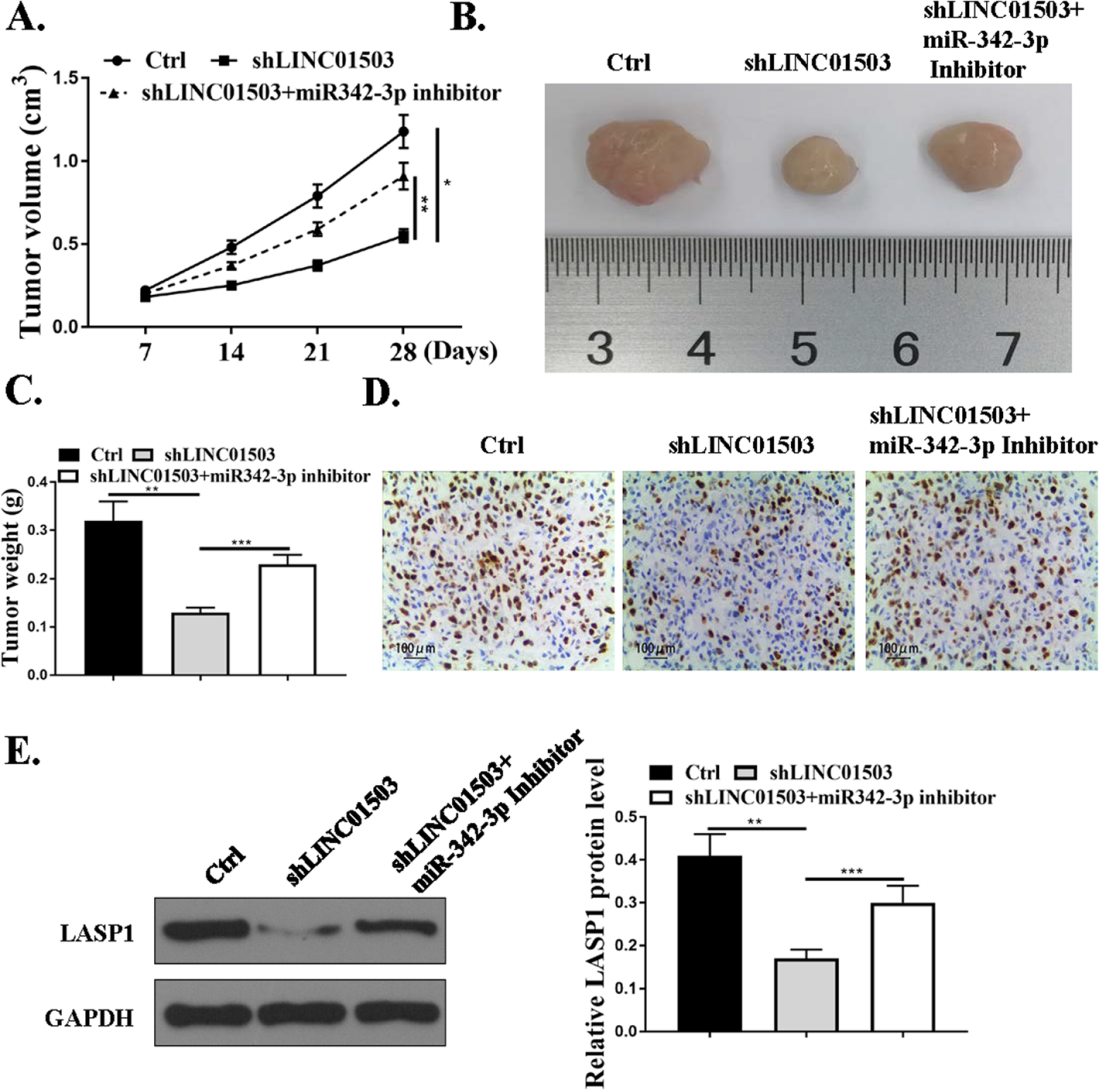
LINC01503/miR-342-3p promotes NSCLC cell tumor growth in vivo. **a.** Tumor volumes were measured every 1 week, and growth curves are shown. **b**. Representative images of mice bearing tumors. **c**. Tumor weight. **d**. Representative images for Ki67 immunostaining of tumor samples from different groups. **e**. The LASP1 protein expression, *N* = 5. *p < 0.001, **p < 0.01, ***p < 0.05

We next injected shLINC01503 knockdown cells, shLINC01503 and miR-342-3p reducing cells, or control cells into nude mice to investigate whether LINC01503/miR-342-3p would influence NSCLC cells tumorigenesis in vivo. The data revealed that tumors grown from cells with the knockdown of LINC01503 were significantly smaller compared with tumors grown from control cells and this effect was attenuated by a miR-342-3p inhibitor (*P*<0.01, *P*<0.001) (Fig. 7a-c). Moreover, tumor samples collected from the LINC01503 knockdown group had fewer Ki67-positive cells, whereas the miR-342-3p inhibitor had more Ki67-positive cells in contrast to the LINC01503 group (Fig. 7d). In addition, shLINC01503 reduced the expression levels of LASP1 and co-transfection with miR-342-3p inhibitor promoted the expression of LASP1 (Fig. 7e). In summary, we proved that LINC01503/miR-342-3p promoted NSCLC cell tumor growth in vivo.

## Discussions

LINC01503 was reported to promote colorectal cancer cell proliferation and invasion by regulating the miR-4492/FOXK1 signaling [23]. In colorectal cancer tissues, they found that LINC01503 was upregulated and miR-4492 was significantly down-regulated [23]. In our experiments, qRT-PCR analysis revealed that the expression of LINC01503 in tumor samples was markedly elevated in contrast to those in the corresponding normal samples. However, the expression levels of miR-342-3p were lower in tumor samples. The expression of miR-342-3p as negatively corelated with that of LINC01503 in NSCLC patients. The expression levels of LINC01503 were also markedly increased and the expression levels of miR-342-3p were reduced in NSCLC cells compared with that in 16HBE cells. Similar to previous published literature, we found that the expression of LINC01503 was up-regulated in NSCLC patients and NSCLC cells, and the expression of miR-342-3p was suppressed.

A recent study reported that LINC01503 promoted cell proliferation, invasion and EMT process in cholangio-carcinoma [24]. We were also interested in the role of LINC01503 in the proliferation, invasion and migration abilities in NSCLC cells. CCK-8 assay and EdU assay revealed that sh-LINC01503 inhibition reduced cell proliferation compared with that of the control cells. Migration and invasion assays revealed a notable reduction in the number of migrated and invaded cells in transfection with LINC01503 shRNA relative to the negative control shRNA group. In consistence with previous studies, we found that LINC01503 promoted proliferation, migration, and invasion of NSCLC cells in vitro.

MiR-342-3p, a well-studied miRNA that has been proved to be a tumor suppressor for the proliferation and invasion of NSCLC [17, 25]. In addition, miR-342-3p was also demonstrated to bind with several different lncRNAs to exert biological functions [26, 27]. From our results, miR-342-3p was predicted to interact with LINC01503 directly. MiR-342-3p markedly inhibited the luciferase activities in cells transfected with the LINC01503-WT but did not affect LINC01503-MUT. RNA pull-down assays pointed out a markedly higher expression levels of LINC01503 in contrast to the assays using NC-bio or hsa-mir-miR-342-3p probes. Sh-LINC01503 reduced the expression levels of miR-342-3p. Moreover, CCK-8 assays and EdU assays revealed that sh-LINC01503 inhibition reduced cell proliferation compared with the control group in cells and this effect was attenuated by co-transfection with miR-342-3p inhibitor. Migration and invasion assays revealed a notable reduce in the number of migrated and invaded cells in transfection with LINC01503 shRNA relative to the negative control shRNA group and this effect was attenuated by co-transfection with miR-342-3p inhibitor. For the first time, we revealed that miR-342-3p was a target of LINC01503 in NSCLC samples and cells.

LASP-1, a cyclic adenosine monophosphate (cAMP) and cGMP-dependent signaling protein, was recently found to play important roles in NSCLC progression. Studies have demonstrated that LASP-1 was notably upregulated and promoted tumor proliferation, invasion and metastasis in multiple malignant tumors, including NSCLC. LASP-1 was reported to be regulated by miR-203 and facilitate tumor proliferation and aggressiveness in human NSCLC [19]. LASP-1 was also reported to promote the malignant phenotype of NSCLC via inducing phosphorylation of the FAK-AKT pathway [28]. From our experiments, LASP1 had a higher expression levels in tumor samples, which was consistent with previous publications [19]. The expression of miR-342-3p was negatively corelated to that of LASP1 in NSCLC patients. LASP1 was also upregulated in NSCLC cells compared with that in 16HBE cells. Moreover, miR-342-3p can directly binding to LASP1. MiR-342-3p mimic reduced cell proliferation compared with the control group in cells and this effect was attenuated by co-transfection with pcDNA-LASP1. PcDNA-LASP1 can also reverse the migration and invasion abilities of miR-342-3p mimic, suggesting that LASP1 and miR-342-3p play opposite roles in NSCLC progression. As far as we know, we are the first to report that the miR-342-3p/LASP1 axis mediated proliferation, migration, and invasion of NSCLC cells in vitro. Besides, we also confirmed the above resutls by in vivo assays.

## Conclusions

LINC01503 as a competing endogenous RNA (ceRNA) regulates NSCLC proliferation and migration via miR-342-3p-LASP1 axis in vitro and in vivo.

## Data Availability

The datasets used and/or analyzed during the current study are available from the corresponding author on reasonable request.

## Abbreviations

NSCLC: Non-small cell lung cancer
lncRNAs: Long non-coding RNAs
LASP1: LIM and SH3 domain protein 1
ceRNA: Competing endogenous RNA
miRNAs: MicroRNAs
FOXM1: Forkhead box protein M1
EdU: 5-ethynyl-29-deoxyuridine
CCK-8: Cell Counting Kit-8
cAMP: Cyclic adenosine monophosphate
